# Potential use of multi-strain synbiotics for improving postnatal head circumference

**DOI:** 10.12669/pjms.346.16107

**Published:** 2018

**Authors:** Ipek Guney Varal, Nilgun Koksal, Hilal Ozkan, Onur Bagci, Pelin Dogan

**Affiliations:** 1 *Ipek Guney-Varal, M.D. Department of Pediatrics, Division of Neonatology, Uludag University Faculty of Medicine, Bursa, Turkey*; 2 *Nilgun Koksal, M.D, Professor of Pediatrics, Department of Pediatrics, Division of Neonatology, Uludag University Faculty of Medicine, Bursa, Turkey*; 3 *Hilal Ozkan, M.D, Associate Professor of Neonatology, Department of Pediatrics, Division of Neonatology, Uludag University Faculty of Medicine, Bursa, Turkey*; 4 *Onur Bagci, M.D. Department of Pediatrics, Division of Neonatology, Uludag University Faculty of Medicine, Bursa, Turkey*; 5 *Pelin Dogan, M.D. Department of Pediatrics, Division of Neonatology, Uludag University Faculty of Medicine, Bursa, Turkey*

**Keywords:** Neurodevelopment, Newborn, Synbiotics

## Abstract

**Background & Objective:**

Preterm infants need nutritional and medical requirements in accordance with the physiologic maturity at birth and maintaining optimal postnatal corporal and cerebral growth is one of the main targets of medical caregivers. However, only a few strategies exist to improve the outcomes of infants in a pathogen-rich and nutrient-poor neonatal intensive care unit environment. In this pilot study, we hypothesize that synbiotics may enhance brain growth, which is reflected indirectly by an increase in head circumference through several signalling molecules.

**Methods:**

A pilot study was conducted in preterm infants with a gestational age of ≤32 weeks and a birth weight of ≤1500 grams at neonatal intensive care unit of Uludag Univeristy Medical Faculty (NICU) for one-year period. Following the randomization of the infants, a prepared commercial synbiotic solution containing multi-combined probiotics and prebiotics was administered enterally to the study group.

**Results:**

The odds of a patient having a lower body weight and head circumference below the 10^th^ percentile were significantly lower in the probiotic group (p=0.001, p=0.03, respectively). Moreover, the infants in the synbiotics group had a more optimal head circumference (between the 50^th^ and 90^th^ percentiles, p=0.001).

**Conclusions:**

Our results show that if we can maintain optimal gut microbiota, we might achieve better neuro-development via the beneficial effects of synbiotics on cytokines, neurotransmitters, and the cellular immunity of the nervous system. Further investigational models are needed to demonstrate the beneficial effects of synbiotics on the central nervous system.

## INTRODUCTION

Preterm infants need nutritional and medical requirements in accordance with the physiologic maturity at birth. The main targets physicians reach for are maintaining optimal postnatal corporal and cerebral growth. In fact, there are only a few approaches to improving the results of infants in the neonatal intensive care unit environment, which is pathogen-rich and nutrient-poor.^1^ Probiotics are micro-organisms that, by colonizing in the gastrointestinal system, create health benefits through improved gut mucosal barrier integrity, bacterial colonization optimization, improved mucosal IgA response, and immunomodulation to the host, which results in an increase in anti-inflammatory cytokines and a reduction in pro-inflammatory cytokines.^2^ Probiotics are becoming more important for preterm newborn nutrition since these infants have frequent gastrointestinal and systemic complications because of the altered microbiota.^3^,^4^ Currently, probiotic use in the neonatal period for preterm infants is generally accepted as a regular element of optimal nutrition.^5^

During the last trimester of pregnancy, important phases of brain growth and maturation occur. Both white and grey matter structures undergo a dramatic increase in volume, with the cerebellum and cortical grey matter showing the highest growth rates.^6^,^7^ Maturation of myelin-producing cells, development of axons, and proliferation and migration of neurons to the cerebral cortex take place in the period between 24 and 40 weeks of gestation.^8^ It is also known that bidirectional signalling exists between the gastrointestinal tract and the brain at the gut-brain axis, which is vital for controlling homeostasis and regulated at the neuronal, hormonal, and immunological levels.^9^ This bilateral interaction may adjust brain function and development with pro- and anti-inflammatory cytokines, chemokines, immune cells, and hormones.^10^ In this pilot study, we hypothesize that synbiotics may enhance brain growth, which is reflected indirectly by an increase in head circumference through these signalling molecules.

## METHODS

A pilot study was conducted on preterm infants with a gestational age of ≤32 weeks and a birth weight of ≤1500 grams at the neonatal intensive care unit of Uludag University Medical Faculty for one-year period. The infants with detected chromosomal abnormalities, previous gastrointestinal system surgery, and infants with a severe sepsis episode were excluded from the study. Alternate randomization was used to enrol the infants in the study; following the randomization of the infants, a prepared commercial synbiotic solution containing the multi-combined probiotics of Lactobacillus rhamnosus (4.1x10^8^cfu), Lactobacillus casei (8.2x10^8^cfu), Lactobacillus plantarum (4.1x10^8^cfu), and Bifidobacterium animalis (4.1x10^8^cfu) (NBL Probiotic®, Nobel, Istanbul, Turkey) together with 383 mg of fructooligosaccharides and 100 mg of galactooligosaccharides as the prebiotic content was administered enterally to the study group.

All infants’ demographic and clinical characteristics were recorded such as birth weight, gestational week, head circumferences, gender, antenatal steroids, mode of delivery and feeding characteristics.

Minimal enteral feeding was started on the first day of life with the infant’s mother’s milk for all patients, and formula feeding was preferred when mother’s milk was not available. The synbiotic supplement was started along with the first enteral feeding and continued until the patient was discharged. The infants who developed abdominal distension, gastric residual, or vomiting during feedings were categorized as having episodes of feeding intolerance and their feeding was suspended. Body weights and head circumferences of the subjects were recorded on a weekly basis on standardized charts. The study was approved by Uludag University Clinical Research Ethics Board. Written consent was obtained from preterm infants’ parents.

Statistical analyses of the data were performed using the Statistical Package for the Social Sciences (SPSS), version 16.0.1 (SPSS Inc., Chicago, IL, USA). All the continuous values were presented as median and mean ± standard deviation, where suitable. The categorical values were presented as numbers and percentages. Chi-square analysis or Fisher’s exact test was used to compare the categorical variables among groups. The Mann-Whitney U-test was used to compare nonparametric variables. Logistic regression analysis was used to identify the factors affecting the outcome. Statistical significance was set at p<0.05.

## RESULTS

A total of 139 patients were included in the study. Of the 110 infants finally analysed, 64% (n=70) were in the group that received probiotics (Group-1), and 36% (n=40) were in the control group (Group-2). Patients were randomized in a 2:1 ration to receive probiotics or not (control group). The demographic and clinical characteristics of the groups are summarized in Table-I. All infants started to be fed within the first hour of life with own mother’s milk as a study protocol. If mother’s milk was not available, preterm formula was given. There was found to be no significant difference between the groups (p=0.108). In Group-1, the initiation of synbiotic supplementation ranged between the 2^nd^ and 7^th^ days postnatal, with a mean of 4.3±1.5 days; patients in Group-1 had a mean of 36.5±12.6 days of synbiotic use. Multivariate analysis was carried out for head circumference of our patients in accordance with birth weight and gestation weeks. Examining birth weight and birth head circumference of groups was not given significant difference. The odds of a patient having a lower body weight and a head circumference below the 10^th^ percentile were significantly lower in the synbiotic group (p=0.001, p=0.03; respectively). Moreover, the infants in the probiotic group had a more optimal body weight and head circumference (between the 50^th^ and 90^th^ percentiles, p=0.001). The feeding characteristics of the study infants are summarized in Table-II. Adverse effect was not observed due to synbiotic supplement in patients.

**Table-I:** Demographic and clinical characteristics of the infants.

	Group 1 (Probiotic group) (n=70)	Group 2 (Control group) (n=40)	P
Gestational age (weeks)	29.7±1.9	29.3±1.7	0.3
Birth weight (gram)	1228±257	1228±249	0.9
Gender, male, n (%)	45 (64)	19 (47)	0.1
Antenatal steroid, n (%)	33 (47)	21 (52)	0.7
Cesarean-section birth, n (%)	58 (82)	31 (77)	0.6

**Table-II:** Weight gain and head circumference at birth and discharge.

		Group 1 Probiotic (n=70)		Group 2 Control (n=40)		P

		n	%	n	%	
Head circumference at birth	<10 p	10	14.3	9	22.5	0.5
	10-50 p	45	64.3	24	60.0	
	50-90 p	15	21.4	7	17.5	
Body weight at birth	<10 p	14	20.0	6	15.0	0.3
	10-50 p	41	58.6	29	72.5	
	50-90 p	15	21.4	5	12.5	
Head circumference at discharge	< 10 p	19	27.1	20	50.0	0.03
	10-50 p	40	57.1	18	45.0	
	50-90 p	11	15.7	2	5.0	
Body weight at discharge	< 10 p	23	32.9	27	67.5	0.001
	10-50 p	33	47.1	12	30.0	
	50-90 p	14	20.0	1	2.5	
*Feeding characteristics*						
Total duration of TPN use, days		13±5		22±11		0.001
Time to full enteral feeding, days		14±3		21±8		<0.001
Feeding intolerance, n (%)		24 (34.3)		31 (79.5)		<0.001

TPN:Total parenteral nutrition.

## DISCUSSION

As recent advances in the science of neonatology have increased the survival of the tiniest of infants, the importance of optimal growth and neurodevelopment has also gained paramount importance. One of the main aims of neonatologists is sustaining this postnatal growth as it would have progressed in the intrauterine period and maintaining optimum neurodevelopment since the time spent in the hospital is the most critical period of postnatal growth. In this pilot study, we used multivariate analysis for head circumference of our patients in accordance with birth weight and gestation weeks. We aimed to show that very low birth weight infants had a shorter time until full enteral feedings, better weight gain, and a larger head circumference at the time of discharge when administered combined multi-strain probiotic and prebiotic supplements, and we demonstrate that synbiotic preparations have beneficial effects on these parameters, the most important of which is the more optimal head circumference growth in.

In preterm infants, microbiota are different from those of healthy term infants, with a gastrointestinal system that is dominated by Proteobacteria initially and a deficiency of detectable Bifidobacterium and Lactobacillus.^11^ Development of gut microbiota is affected by the mode of delivery, colonization from the surrounding environment, use of antibiotics, and feeding characteristics. Breastmilk is a rich source of mutual microorganisms, which are necessary for the maintenance of healthy microbiota. Besides regulating microbiota directly and indirectly, breastfeeding is a source of multiple neuroactive agents or their precursors such as tryptophan and can also affect the central nervous system.^12^ In animal studies, administration of Bifidobacterium infants enhanced the plasma concentration of tryptophan, suggesting that normal microbiota can affect the precursor pool for serotonin (5-HT).^13^,^14^ In addition to optimal development of the neuroendocrine system, head growth is a proxy measure of brain growth, which can be assessed by means of magnetic resonance imaging.^15^ In our study, we demonstrate that infants on synbiotics needed less time to reach full enteral feedings, which was in accordance with the previous literature. However, we believe our novel finding that the infants on synbiotics had a larger head circumference upon discharge from the hospital deserves attention.

Prebiotic oligosaccharides are a group of nutritional components that may provide benefits to the developing preterm brain. Prebiotic oligosaccharides show the potential to improve the infant’s intestinal microbiota by enhancing the growth of Bifidobacterium, which may decrease the load of potentially pathogenic micro-organisms in the sequence in the gut.^16^ In a recent study, despite supplementation of prebiotics, there was no improvement in neuro-developmental outcomes, and lower Bifidobacteria colonization correlated with lower neuro-developmental outcomes in preterm infants.^17^ This bifidogenic effect contributes to the survival of probiotic micro-organisms in the host and regulates an immunologic interaction in the neuronal network.^18^ Our synbiotic combination also contained a prebiotic compound, which might further explain the improved neurological development of premature infants, reflected by an optimal head circumference.

Nowadays, it is well known that normal gut microbiota are important for the optimal neurological and behavioural development of the human organism.^19^ In recent reviews, effects of antimicrobial properties of different probiotics on gut immune system and homeostasis by enhancing the gut epithelial barrier and modulating mucus production indicated.^20^ However, it is not well established which nutrients and to what extent nutrient components have an effect on neurological maturation. Our results show that if we can maintain optimal gut microbiota, we might achieve better neuro-development via the beneficial effects of synbiotics on cytokines, neurotransmitters, and the cellular immunity of the nervous system. Further investigational models are needed to demonstrate the beneficial effects of synbiotics on the central nervous system.

### Limitations of the study

First one was limited number of patients while second was not assessing the correlation between head circumference and neurological development at two years of age monitoring of our patients. However, we are evaluating our study in this respect.

### Authors’ Contribution

**IGV:** Conceived the original idea, initial and final draft, data supervision, writing and critical review of the manuscript.

**NK:** Designed the study, data analysis and interpretation.

**HO:** Did data analysis and interpretation.

**OB:** Designed tables with text data.

**PD:** Did data collection and added references text.
